# Wide Temperature Zero Thermal Expansion in Al Matrix Composites with High Thermal Conductivity

**DOI:** 10.1002/advs.75748

**Published:** 2026-05-19

**Authors:** Jinrui Qian, Feixiang Long, Yiqing Liu, Longlong Fan, Mingqing Liao, Qilong Gao, Hao Lu, Dexiang Gao, Le Kang, Yuzhu Song, Xiuzhu Han, Yue Sun, Naike Shi, Chang Zhou, Jun Chen

**Affiliations:** ^1^ State Key Laboratory for Advanced Metals and Materials Department of Physical Chemistry University of Science and Technology Beijing Beijing China; ^2^ State Key Laboratory of Tropic Ocean Engineering Materials and Materials Evaluation Hainan University Haikou China; ^3^ Department of Physical Chemistry University of Science and Technology Beijing Beijing China; ^4^ Institute of High Energy Physics Chinese Academy of Sciences Beijing China; ^5^ School of Materials Science and Engineering Jiangsu University of Science and Technology Zhenjiang China; ^6^ Key Laboratory of Materials Physics of Ministry of Education and School of Physics and Microelectronics Zhengzhou University Zhengzhou China; ^7^ Spallation Neutron Source Science Center Dongguan China; ^8^ School of Advanced Materials Innovation University of Science and Technology Beijing Beijing China

**Keywords:** metal matrix material, negative thermal expansion, phase transition, strain engineering

## Abstract

Lightweight Al composites enhanced with negative thermal expansion (NTE) materials exhibit ultra‐low temperature sensitivity, offering substantial promise for high‐precision and aerospace applications. However, most strong NTE reinforcements driven by abrupt phase transitions tend to present a narrow operation window, limiting the realization of wide‐range zero thermal expansion (ZTE) behavior in the composites. Herein, this critical challenge is addressed in the Zn_1.6_Mg_0.4_P_2_O_7_/Al composites through a strain engineering strategy. Under the Al matrix‐induced compressive strain, Zn_1.6_Mg_0.4_P_2_O_7_ exhibits a broad uniform NTE response spanning 80°C, contrasting sharply with its inherent narrow NTE temperature window (20°C). As a result, the composite with 35 vol.% Zn_1.6_Mg_0.4_P_2_O_7_ achieves high‐performance ZTE (0.90 ppm/°C) across 25–80°C. Meanwhile, due to the high content of the Al matrix with high thermal conductivity, this ZTE material exhibits excellent thermal conductivity (80.9 W·m^−1^·K^−1^) compared with the ZTE alloy Invar (12.8 W·m^−1^·K^−1^). In situ neutron powder diffraction, Raman spectroscopy, and X‐ray diffraction characterizations demonstrate that the wide and gradual volume shrinkage in the NTE phase is attributable to strain‐driven structural transformations. This work presents a simple yet effective approach for homogenizing the performance of NTE materials and holds great potential for facilitating the practical applications of specialized NTE materials.

## Graphical abstract

Strain engineering strategy is implemented on the negative thermal expansion (NTE) phase Zn_1.6_Mg_0.4_P_2_O_7_ within composites, where Al matrix‐induced compressive strain drives uniform polyhedral and unit cell evolution, thereby enabling broad and consistent thermal compensation efficacy and obtaining good thermal performance. This work validates the feasibility of strain engineering for achieving high‐performance zero thermal expansion in high‐coefficient‐of‐thermal‐expansion matrix composites.

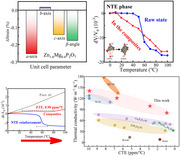

## 1 Introduction

Zero thermal expansion (ZTE) materials retain dimensional stability within a specific temperature range, enabling their critical applications in aerospace, microelectronics, precision instruments, and related fields [1, 2, 3]. Aluminum (Al) matrix composites, featuring low density, high thermal conductivity (*λ*), and high specific strength, enjoy widespread applications in modern industrial scenarios [4, 5, 6]. Unfortunately, this prominent lightweight engineering material faces substantial challenges in meeting stringent dimensional thermal stability requirements, primarily due to its high coefficient of thermal expansion (CTE) of 20–24 ppm/°C [7, 8, 9]. As demands for engineering in extreme environments continue to grow, the limitation of developing excellent Al matrix composites with low temperature sensitivity and ZTE performance in particular must be critically addressed.

Preparing Al matrix composites using low‐thermal‐expansion (LTE) materials, such as SiC and AlN, represents a widely used approach for reducing the CTE [10, 11, 12, 13]. However, as these reinforcements exhibit positive thermal expansion (PTE) characteristics, achieving ultra‐LTE in the composites remains a major challenge [14]. Negative thermal expansion (NTE) materials, which contract upon heating, offer a viable pathway for designing ultra‐low or zero thermal expansion metal matrix composites [15, 16, 17, 18, 19]. Over the past few decades, numerous ZTE metal matrix composites have been reported, including ZrW_2_O_8_/Al and La(Fe, Si, Co)_13_/Cu composites [20, 21, 22]. Given the high CTE of Al, conventional NTE reinforcements (e.g., ZrW_2_O_8_ and PbTiO_3_) require high volume fractions (>70 vol.%) to achieve ZTE in Al matrix composites. However, most NTE materials suffer from low thermal conductivity and inherent brittleness, which severely degrade the overall performance of composites [20, 23, 24, 25, 26, 27, 28, 29]. Notably, certain giant NTE reinforcements with first‐order phase transitions (e.g., La(Fe, Si, Co)_13_, Mn_3_Zn_1‐_ *_x_* Sn*_x_* N) can achieve ZTE in Al matrix composites at relatively low contents [30, 31, 32]. Nevertheless, these NTE materials usually exhibit a narrow NTE temperature window, which impedes their practical applications in scenarios involving wide‐ranging temperature fluctuations. Despite advances in controlling CTE over a wide temperature range via microstructure design and multiphase hybrids, developing Al matrix composites with ZTE using minimal NTE phases remains challenging [33, 34, 35]. Thus, an innovative design strategy is urgently required to develop Al matrix composites with good ZTE performance, thereby facilitating their broader application in low‐temperature‐sensitivity scenarios.

Strain engineering, enabled via interface interactions, modulates the crystal structure of materials, emerging as a promising strategy to tailor the overall physical properties of composites [36, 37, 38, 39, 40]. In Al matrix composites, the significant CTE mismatch between the NTE phase and Al causes severe stress/strain concentration at the interface upon entering the NTE temperature window [41, 42, 43]. For instance, during the fabrication of the ZrW_2_O_8_/Al composite, thermal mismatch stresses of up to 600 MPa arise within the composite owing to opposing lattice evolution upon cooling [20]. Notably, the NTE materials with distinct axial ratios (e.g., PbTiO_3_, Zn_2_P_2_O_7_) exhibit high sensitivity to lattice strain [44, 45, 46, 47]. The NTE reinforcement under the strain effect constitutes a key attribute for regulating the thermal expansion performance of composites. For instance, the large lattice strain introduced by PbO in PbTiO_3_ elongates its *c*‐axis at room temperature, which increases the volume difference between the room‐temperature phase and the high‐temperature phase [36]. Similarly, a hydrostatic pressure of 250 MPa enhances the NTE effect of Cu_2_P_2_O_7_ by 47.5% relative to its pristine state under ambient pressure [45]. In Cu_2_P_2_O_7_/Al composites, the relatively high residual stress (165–243 MPa) not only enhances the NTE effect but also shifts the NTE temperature range closer to room temperature [48, 49]. This strategy can also be extended to magnetic phase‑transition materials. Strain engineering effectively strengthens the NTE effect in hexagonal (Ni_1‐_ *_x_* Fe*_x_*)_1‐δ_S and broadens its working temperature window [50]. Therefore, the structural phase transition in NTE materials driven by strain from the matrix would be expected to achieve a strengthened NTE effect, thereby enabling the design of Al matrix composites with excellent ZTE performance.

Herein, we demonstrate that strain derived from the Al matrix homogenizes the abrupt structural phase transition of the NTE reinforcement. Specifically, the Al matrix composite incorporating a low content (35 vol.%) of the NTE phase achieves excellent ZTE performance over the 25–80°C range. Meanwhile, a high thermal conductivity (80.9 W·m^−1^·K^−1^) is presented in this ZTE material. High‐resolution transmission electron microscopy (HR‐TEM) confirms a well‐coherent interface between the NTE phase and Al. The regulated phase transition process of the NTE reinforcement upon heating under pressure is elucidated via Raman spectroscopy, finite element analysis, X‐ray diffraction (XRD), and neutron powder diffraction (NPD). Furthermore, the correlation between local structure and properties is established by integrating structural extraction from diffraction patterns with first‐principles density functional theory (DFT) calculations. This study supports the development of novel LTE/ZTE Al matrix composites and presents a transferable strategy for other composite systems.

## 2 Results and Discussion

### 2.1 Phase Structure

**FIGURE 1:**
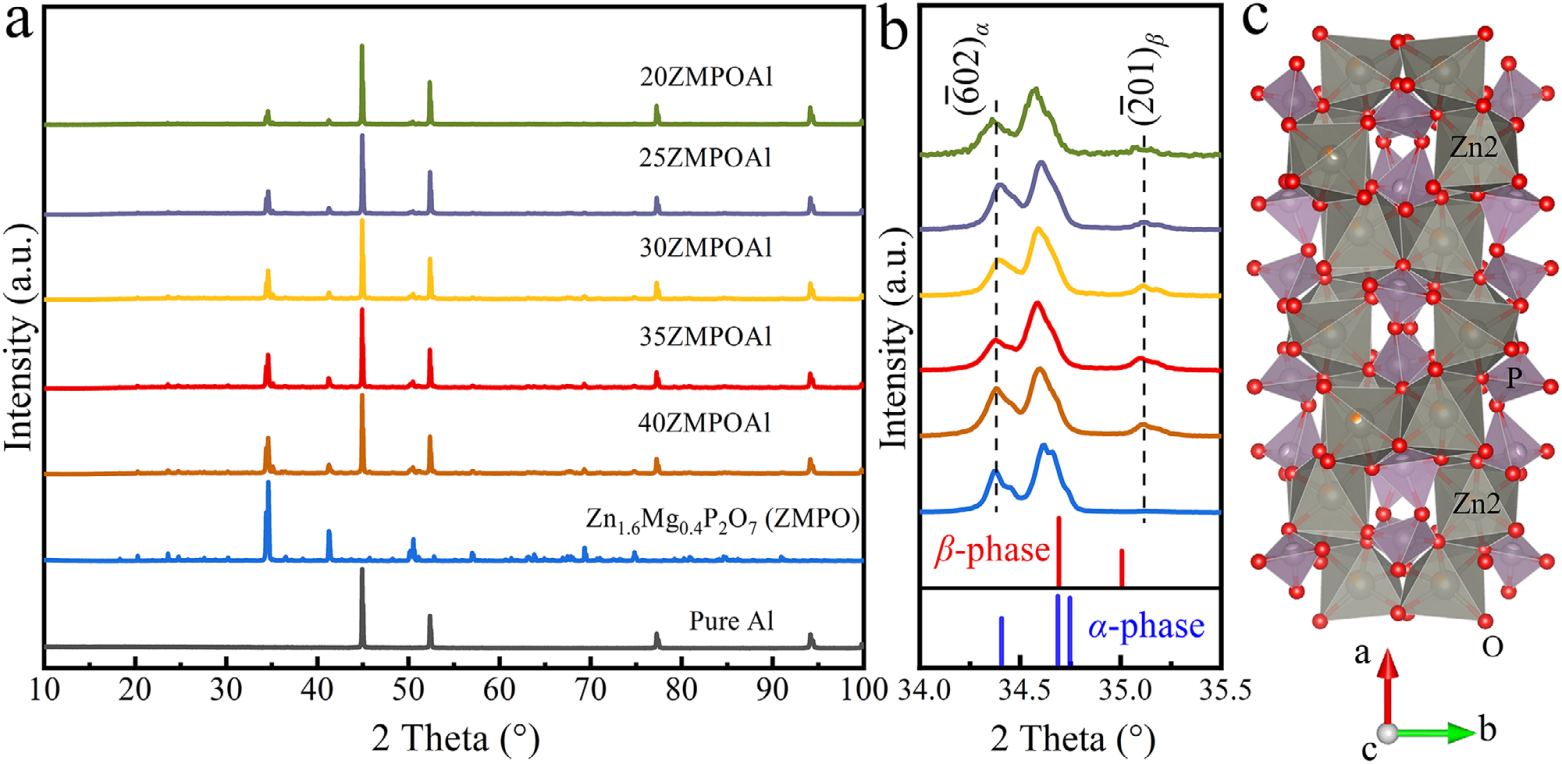
Phase composition of the ZMPOAl composites. (a) XRD patterns of the ZMPOAl composites at room temperature. (b) Magnified view of the ZMPO diffraction peaks in the 34.0–35.5° range from (a), compared with PDF #72‐1702 (*α*‐Zn_2_P_2_O_7_) and #73‐1648 (*β*‐Zn_2_P_2_O_7_). (c) Crystal structure of the *α*‐ZMPO; gray spheres represent Zn/Mg atoms, red spheres represent O atoms, and purple spheres represent P atoms.

Zn_1.6_Mg_0.4_P_2_O_7_ (ZMPO) was selected as a reinforcement to investigate the strain engineering strategy for enhancing the NTE effect, owing to its high axial ratio (*a*/*b* = 2.426) and strong NTE behavior induced by structural transitions [51]. In this work, ZMPO/Al composites with varying ZMPO volume fractions (20%, 25%, 30%, 35%, and 40%) were designed and fabricated via spark plasma sintering (SPS) to effectively offset the high PTE of the Al matrix. These composites were denoted as “20ZMPOAl”, “25ZMPOAl”, “30ZMPOAl”, “35ZMPOAl”, and “40ZMPOAl”, respectively. The XRD spectra of the composites (Figure 1a) show that no detectable peaks of other phases can be found, suggesting a well‐controlled interfacial structure during fabrication. Structure refinement of the XRD pattern for raw ZMPO powder (see Figure S1) confirms a dual‐phase composition of low‐temperature *α*‐ZMPO and high‐temperature *β*‐ZMPO, consistent with previous reports [32, 51]. Notably, shifts in peak positions are barely noticeable in Figure 1b, but there are significant changes in peak intensities. The composites show a stronger *β*‐ZMPO ($\bar{2}$01) diffraction peak but a weaker *α*‐ZMPO ($\bar{6}$02) peak than the raw powder. Typically, the *a*‐axis and *c*‐axis lengths of *β*‐ZMPO (space group *C*2/*m*, Figure S2) are 1/2 and 1/3 those of *α*‐ZMPO (space group *I*2/*c*, Figure 1c), respectively [51]. During the *α*‐to‐*β* phase transition, the intensity of the *α*‐ZMPO ($\bar{6}$02) peak diminishes, whereas that of the *β*‐ZMPO ($\bar{2}$01) peak increases. The refined results of the ZMPO in raw powder (see Supplementary Table S1) and 35ZMPOAl (Table S2) demonstrate that while the NTE phase structure is stable, the *α*‐ZMPO phase content decreases from 97.1 wt.% to 85.5 wt.%. Additionally, the *a*‐axis, *c*‐axis, and *β*‐angle of the ZMPO in the composite decrease, while the *b*‐axis remains nearly unchanged. Specifically, the unit cell volume of the ZMPO (Table S3) shows a reduction from 1452.24 Å^3^ in raw powder to 1448.14 Å^3^ in the composite. The pronounced difference in structural parameters suggests that the ZMPO exhibits a compressed lattice in the composites. As a result, this change regulates related NTE performance.

### 2.2 NTE/Matrix Interface of Composites

**FIGURE 2:**
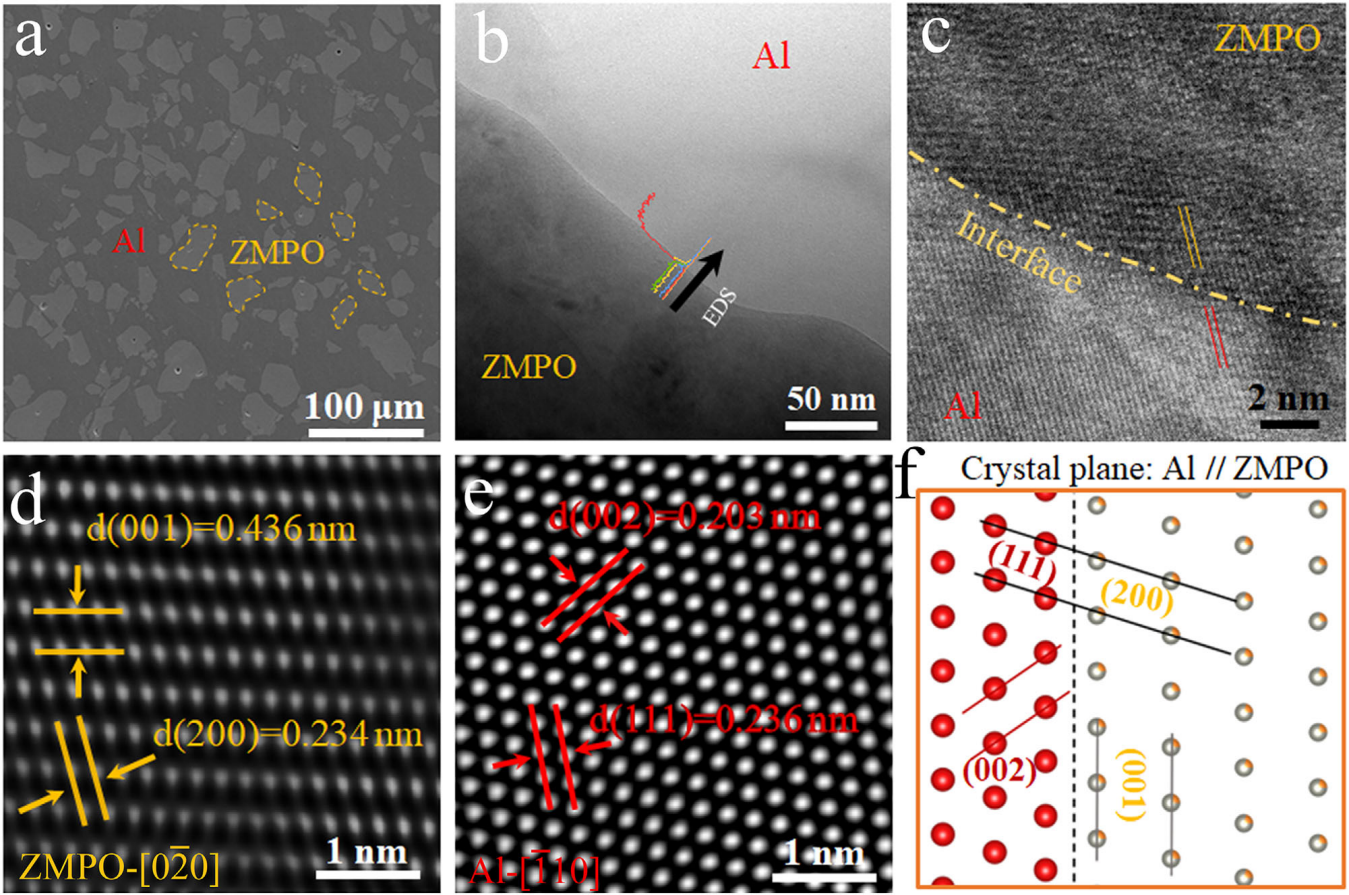
Interface structure of the ZMPOAl composites. (a) SEM image of the 35ZMPOAl composite. (b) Bright‐field TEM image of the composite interface; the corresponding EDS line scan across the interface is provided in Figure S4. (c) HR‐TEM image of the ZMPO/Al interface. (d,e) Corresponding IFFT profiles of the ZMPO (d) and Al (e) regions. (f) Schematic illustration of the atomic configuration near the interface; red spheres represent Al atoms, and gray spheres represent Zn/Mg atoms. For clarity, P and O atoms are omitted in (f).

To investigate the phase distribution and interfacial bonding of the composites, scanning electron microscopy (SEM) and transmission electron microscopy (TEM) were employed for microstructural characterization. As illustrated in Figure 2a, the ZMPO phases, manifested as light‐colored “islands,” are uniformly distributed throughout the continuous dark‐colored Al matrix. Further, bright‐field TEM images of the two‐phase interface (Figure 2b) reveal a smooth and continuous ZMPO/Al interface without nanogaps or interfacial compounds. Meanwhile, Figure S3a,b display the selected area electron diffraction (SAED) patterns obtained from the upper‐right white region and lower‐left gray region in Figure 2b, which are indexed as the Al matrix and ZMPO reinforcement, respectively. Furthermore, the nanoscale line‐scan analysis across the interface (Figure S4) reveals negligible elemental diffusion between ZMPO and Al. Elemental mapping (Figure S5) of the region in Figure 2a further validates the homogeneous distribution of elements within ZMPO particles and the Al matrix, indicating no formation of impurity phases during fabrication, consistent with XRD results (Figure 1a). To clarify the interfacial bonding mechanism, HR‐TEM images of the interface were acquired. As shown in Figure 2c, ZMPO exhibits well‐ordered atomic layers with interplanar spacings comparable to those of the Al matrix. Figure 2d,e present inverse fast Fourier transform (IFFT) images of ZMPO and Al near the interface, respectively. For ZMPO along the [0$\bar{2}$0] zone axis, the interplanar spacings of the (200) and (001) crystal planes are measured to be 0.234 and 0.436 nm, respectively. Importantly, the interplanar spacing of the (111) plane in Al (0.236 nm) closely matches that of the (200) plane in ZMPO. Figure 2f shows the schematic illustration of the atomic configuration near the interface. The lattice mismatch ratio between ZMPO and Al is only 0.8%, which accords with the coherent interface. This well‐bonded interface not only facilitates thermal stress transfer but also maximizes the thermal expansion compensation effect of ZMPO. Moreover, the stable elemental composition of the composite validates that the anomalous ZMPO phase structure in XRD (Figure 1b) stems from compressive strain.

### 2.3 Thermal Expansion Performance of ZMPOAl Composites

**FIGURE 3:**
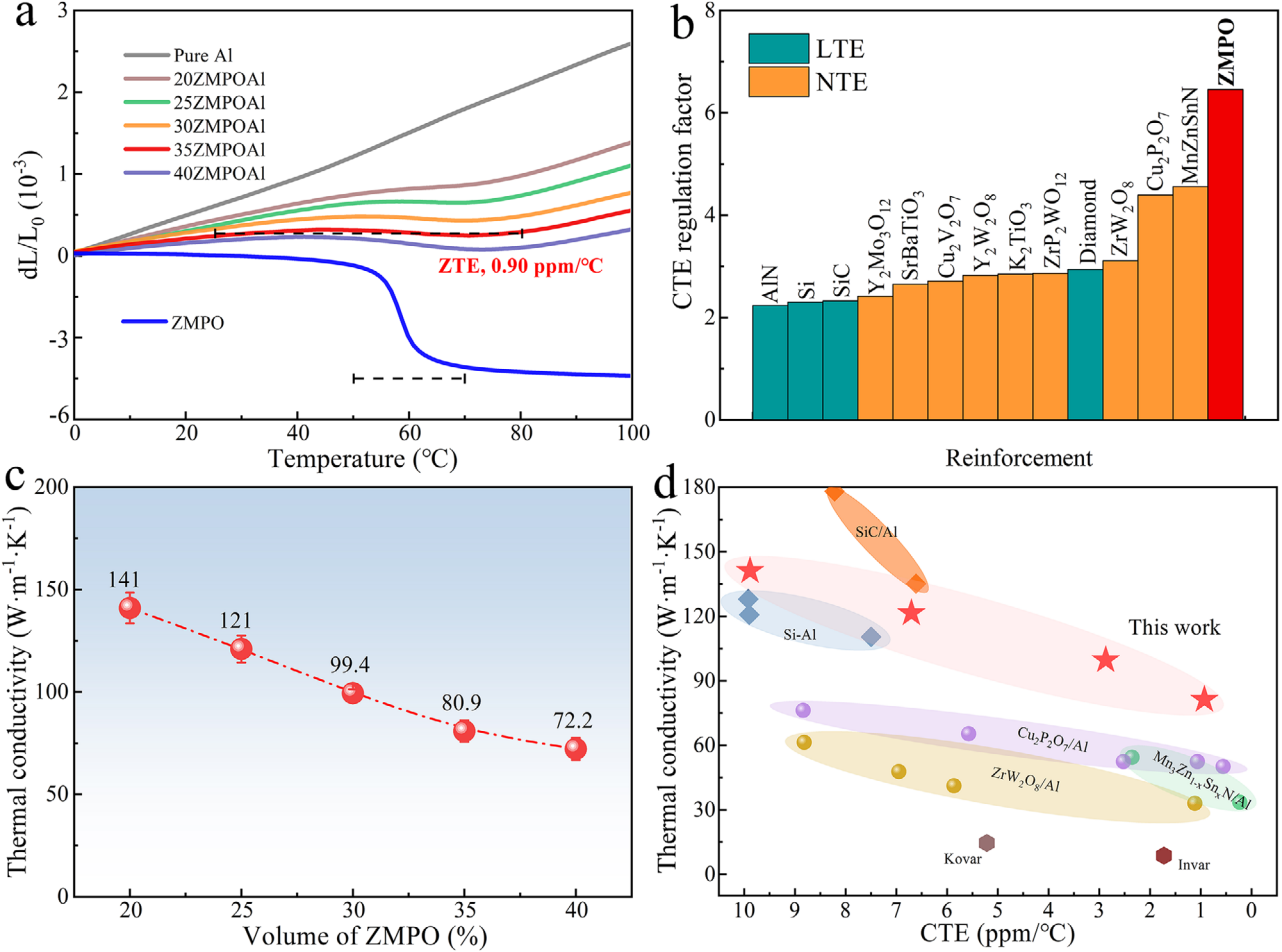
Thermal expansion behavior of the composites. (a) Linear thermal expansion curves of the composites. (b) Comparison of the CTE regulation factor between LTE and NTE reinforcements [13, 20, 22, 23, 24, 25, 26, 27, 28, 29, 34, 52, 53]. (c) Thermal conductivity of the composites at room temperature. (d) Comparison of the CTE‐thermal conductivity for the composites in this work with other LTE/ZTE alloys and Al matrix composites [22, 34, 42, 54, 55, 56, 57, 58, 59, 60].

The thermal expansion of the composites results from the combined effects of phase‐transition‐induced NTE in ZMPO and PTE driven by non‐harmonic atomic vibrations in Al. Figure 3a presents the linear thermal expansion (dL/L_0_) of the composites measured from 0°C to 100°C during heating. The pure Al matrix exhibits a high linear coefficient of thermal expansion (*α*_L_) of 23.5 ppm/°C. In contrast, ZMPO displays NTE behavior with an *α*_L_ of −57.0 ppm/°C over the 30–90°C range. Within the NTE temperature range, all composites exhibit a consistent decrease in the CTE relative to pure Al due to the strong thermal expansion compensation of ZMPO. Among them, the 35ZMPOAl composite exhibits ZTE behavior, with an *α*_L_ of 0.90 ppm/°C from 25°C to 80°C. Notably, the ZTE temperature range (55°C) of this composite is over three times wider than that of previously reported conventional phase‐transition Mn_3_Zn_0.7_Sn_0.3_N‐based Al matrix ZTE composites (15°C) [52]. Tunable CTE values of the composites are achievable by adjusting the ZMPO/Al composition. Specifically, the *α*_L_ values of 20ZMPOAl, 25ZMPOAl, 30ZMPOAl, and 40ZMPOAl over the 25–80°C range are 9.88, 6.69, 2.85, and −1.49 ppm/°C, respectively. Furthermore, 35ZMPOAl was subjected to 100 thermal shock cycles (Figure S6) between liquid nitrogen (−160°C) and hot oil (200°C). The *α*_L_ remained nearly unchanged, revealing good thermal fatigue resistance. Further measurements of *α*_L_ during cooling indicate that the onset temperature of the effective NTE range for ZMPO shifts from 80°C to 42°C in the 35ZMPOAl composite (Figure S7). As a result, a thermal hysteresis of 38°C is observed in the thermal compensation behavior of ZMPO between heating and cooling processes. This hysteresis can be ascribed to compressive stress exerted on ZMPO particles during cooling, which restrains the phase transition from *β*‐ZMPO to *α*‐ZMPO [50]. Nevertheless, this transition still occurs over a wide temperature interval. Importantly, the intrinsic thermal expansion compensation temperature range of raw ZMPO is confined to 50–70°C, in contrast to the broad, uniform range of 25–80°C achieved in the composites. The enhanced NTE performance of ZMPO within the composites is strongly correlated with structural modifications induced by compressive strain.

To assess thermal expansion suppression of the ZMPO, the CTE regulation factor (ΔCTE/V_NTE_) [48] was calculated, quantifying the thermal compensation efficiency of various reinforcements in LTE/ZTE composites (Figure 3b). Notably, NTE reinforcements exhibit substantially higher CTE regulation factors than conventional LTE materials, highlighting their superior potential for realizing LTE or ZTE behavior in the composites. Specifically, the CTE regulation factor of the ZMPO is twice that of the typical NTE reinforcement ZrW_2_O_8_ and three times that of the commercially used LTE reinforcement SiC, demonstrating exceptional thermal expansion compensation capability.

Owing to the intrinsic low thermal conductivity of numerous NTE materials, achieving ZTE Al matrix composites with thermal conductivity values above 50 W·m^−1^·K^−1^ remains a great challenge [7, 22, 34]. Notably, the optimal CTE modulator of the ZMPO effectively ensures the high thermal conductivity of the composites. As illustrated in Figure 3c, the thermal conductivity (Table S4) of the composites exhibits a positive correlation with Al content. For example, the *α*_L_ of 25ZMPOAl matches well with that of well‐established semiconductors such as Si (4.1 ppm/°C) and GaAs (5.8 ppm/°C). Concurrently, its superior thermal conductivity of 121 W·m^−1^·K^−1^ effectively governs the heat exchange process between the composite and chip components. To further clarify the high thermal conductivity mechanism of the composite, the experimentally measured thermal conductivity is compared with predictions derived from classical theoretical models for composite thermal conduction (Figure S8 and Table S5). Compared with the Maxwell model, the Hasselman–Johnson (H–J) model incorporates interfacial thermal resistance (Table S6) and accounts for the particle size of the ZMPO reinforcement (20.68 µm). Interfacial thermal conductance calculations demonstrate that the ZMPO exhibits a smaller acoustic velocity mismatch and favorable acoustic impedance matching with the Al matrix. This leads to higher phonon transmittance and lower interfacial thermal resistance (0.449×10^−8^ m^2^·K·W^−1^). However, the intrinsically ultra‐low thermal conductivity (0.379 W·m^−1^·K^−1^) of the ZMPO shifts the thermal transport bottleneck from the interface to the reinforcing phase. Consequently, the predictions from the H–J and Maxwell models show good consistency. The experimental thermal conductivity of the composite is lower than the theoretical predictions, which may originate from incomplete densification during fabrication. Thermal transport in the composite is dominated by the highly conductive aluminum matrix. No elemental interdiffusion or interfacial reaction occurs between the reinforcement and the matrix, and the aluminum matrix maintains a continuous network. These factors collectively contribute to the excellent thermal conductivity of the composite.

The CTE and thermal conductivity for the composites with other LTE/ZTE alloys and Al matrix composites were compared to show the robust NTE response by this strategy (Table S7). It is found from Figure 3d that traditional LTE reinforcements (SiC, Si) typically yield a CTE below 7.5 ppm/°C in Al matrix composites, even at a ceramic content as high as 50 vol.%. By contrast, NTE alloys suffer from inherent phonon–electron coupling, which poses a major obstacle to achieving high thermal conductivity while maintaining LTE or ZTE. [34] Interestingly, this work achieves ZTE Al matrix composites by incorporating strong NTE materials at low content levels, demonstrating excellent thermal performance in NTE/Al composites and ZTE materials. For instance, the ZTE 35ZMPOAl composite exhibits a lower density of 3.19 g/cm^3^ and superior thermal conductivity of 80.9 W·m^−1^·K^−1^ compared with the well‐established Invar alloys (8.1 g/cm^3^, 12.8 W·m^−1^·K^−1^). Furthermore, ZMPO exhibits an elastic modulus (125 GPa) close to that of the Al matrix. This attribute endows the composite with good processability, as confirmed by the compressive stress–strain curve (Figure S9) [22]. In particular, 35ZMPOAl presents favorable thermal properties together with compressive yield strength of 111 MPa and a compressive strain of 21.5%. The integration of these properties is critical for the fabrication of high‐precision, high‐performance ZTE components.

### 2.4 Structure Evolution of ZMPO Under Pressure

**FIGURE 4:**
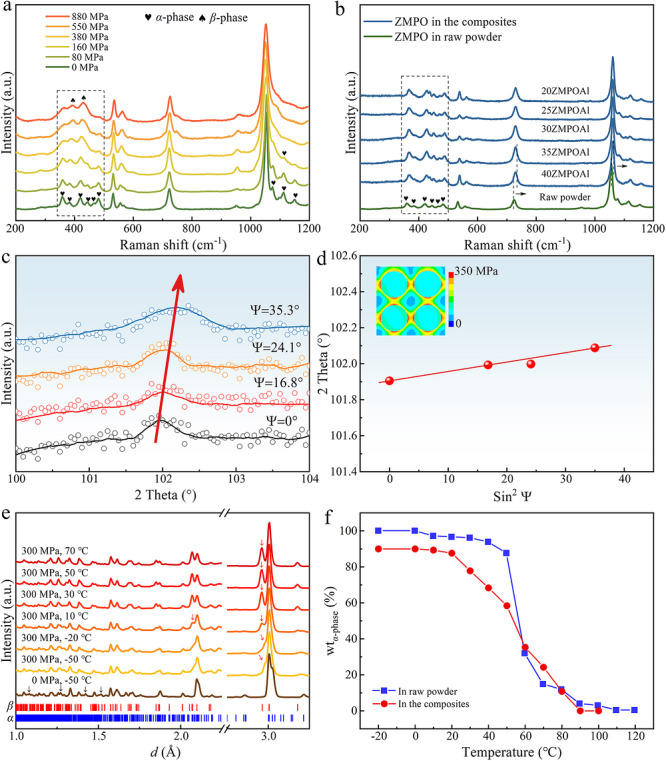
Phase evolution of ZMPO under pressure. (a) Pressure‐dependent Raman spectra of raw ZMPO powder. (b) Raman spectra of ZMPO particles in the composites; the inset shows stress distribution mapping of ZMPO in the 35ZMPOAl composite. (c) GIXRD patterns obtained with different instrumental *ψ* values from 0° to 35.3°. (d) Linear fitting plots of 2*θ*‐sin^2^ *ψ* for determining the stress value; the inset shows stress distribution mappings of ZMPO in the 35ZMPOAl composite. (e) Temperature‐dependent NPD patterns of raw ZMPO powder at 0 and 300 MPa. (f) Mass fractions of *α*‐ZMPO and *β*‐ZMPO in the composites and raw powder.

Investigating the phase evolution of raw ZMPO powder under pressure is crucial for elucidating the origin of its wide, uniform NTE behavior. To this end, pressure‐dependent Raman spectroscopy was employed to systematically characterize the effect of compressive stress on the local structure of the ZMPO (Figure 4a). Below 80 MPa, the Raman peaks of ZMPO remain stable, indicating a single *α*‐phase and structural stability [61]. As pressure increased further, peak broadening was observed in the 300–500 cm^−1^ range, while the *α*‐phase characteristic peak at 1115 cm^−1^ gradually decreased in intensity. Ultimately, all *α*‐phase‐associated characteristic peaks vanished at 550 MPa. These results confirm that ZMPO undergoes a structural transition within the pressure range of 80–550 MPa. Zn_2_P_2_O_7_ is the parent phase of Mg‐substituted ZMPO and shows pressure‐induced NTE and a corresponding structural phase transition. At 1.39 GPa, Zn_2_P_2_O_7_ transforms fully into a single *β*‐phase. Notably, temperature and pressure drive this transition through analogous mechanisms. By comparison, ZMPO undergoes a phase transition at a significantly lower pressure. The structural phase transition in ZMPO driven by low‐pressure conditions lays the foundation for leveraging strain engineering to modulate thermal expansion. Furthermore, Raman spectroscopy was also performed on ZMPO particles within the composites. As shown in Figure 4b, ZMPO in composites exhibits Raman peaks similar to those of raw ZMPO. Nevertheless, the *α*‐phase peak intensity decreases in the composites, accompanied by peak broadening in the 300–500 cm^−1^ range. The Raman peaks at 1061 and 731 cm^−1^ in the composites show a significant blue shift compared to the raw powder, suggesting shortened bond lengths in ZMPO [62].

The unusual thermal expansion behavior described above is closely associated with internal stress generated during composite fabrication. Further evidence for thermal misfit stress in the composite was obtained using grazing‐incidence X‐ray diffraction (GIXRD) at various tilt angles *ψ* (Figure 4c). With increasing *ψ* from 0° to 35.3° (probing deeper regions), the diffraction peak shifts gradually toward higher angles. According to Bragg's law, this shift confirms a progressively larger lattice contraction, indicating that the ZMPO in the composite experiences compressive strain. The misfit stress was quantified using the linear relationship between 2*θ* and sin^2^ *ψ* (Figure 4d). Notably, a positive slope confirms that the ZMPO is under compressive stress, yielding a value of −108 MPa. Thermal stress mismatch in the 35ZMPOAl composite was further quantified via numerical simulation (inset of Figure 4d), revealing an average compressive stress of ∼160 MPa in ZMPO. The discrepancy mainly arises because GIXRD probes stress near the sample surface, while finite‐element simulation reflects the bulk‐averaged stress in the composite. This stress magnitude surpasses the critical threshold necessary for initiating the phase transition of ZMPO. As a result, the compressive stress not only induces the retention of the high‐temperature *β*‐phase at room temperature (Figure 1b) but also leads to a reduced unit cell volume (Tables S1 andS2).

To further elucidate the combined effects of temperature and pressure on the phase behavior of ZMPO, high‐precision NPD measurements under pressure were performed. For raw ZMPO (without pressure application) tested by in situ temperature‐dependent XRD (Figure S10a), the distinct ($\bar{2}$01) diffraction peak of *β*‐ZMPO emerges at 50°C and stabilizes at 70°C with increasing temperature. Upon pressurization, all in situ variable‐temperature NPD patterns of ZMPO can be indexed to either the monoclinic *α*‐phase or *β*‐phase structure (Figure 4e). At −70°C and 0 MPa, ZMPO exhibits a pure *α*‐phase structure. Interestingly, upon pressurization, the diffraction peaks at 2.1 and 3.0 Å broaden (red arrow), while the low‐interplanar‐spacing diffraction peaks corresponding to the low‐symmetry *α*‐phase (black arrow) partially disappear. These changes indicate that pressure drives the transition of ZMPO from the low‐symmetry *α*‐phase to the high‐symmetry *β*‐phase. Furthermore, distinct *β*‐phase characteristic peaks at 2.07 Å and 2.96 Å are clearly visible at 10°C under 300 MPa, and their intensities stabilize by 50°C. This confirms that the ZMPO undergoes an earlier phase transition from −20°C to 50°C under 300 MPa pressure compared to 0 MPa pressure conditions (40–70°C).

To further validate the uniform thermal compensation effect of ZMPO reinforcements, the 35ZMPOAl composite was subjected to detailed in situ temperature‐dependent XRD characterization (Figure S10b). ZMPO within the composite retains a stable first‐order phase transition behavior. Notably, the characteristic ($\bar{2}$01) diffraction peak of *β*‐ZMPO is clearly detectable at 20°C but vanishes completely by 70°C. This thermally induced peak disappearance is determined to be the pivotal factor enabling the broad ZTE performance range of the composite. Given the difficulty in distinguishing overlapping diffraction peaks, structural refinement of the data in Figure S10 was performed to clarify in detail the phase composition evolution with increasing temperature of ZMPO in both raw powder and composites (Table S8). As shown in Figure 4f, raw ZMPO undergoes phase transition over the 0–110°C range, with a marked formation of *β*‐ZMPO between 50°C and 70°C. Significantly, ZMPO in the composite initiates phase transition at 0°C and completes it by 90°C, with a more homogeneous transformation process occurring between 20°C and 90°C. This temperature range aligns well with the ZTE region in the composite (Figure 3a). These results confirm that pressurization advances and homogenizes the phase transition of ZMPO. The inherent compressive strain in the composite leverages this regulated phase transition process, enabling more effective compensation for the high PTE of the Al matrix.

**FIGURE 5:**
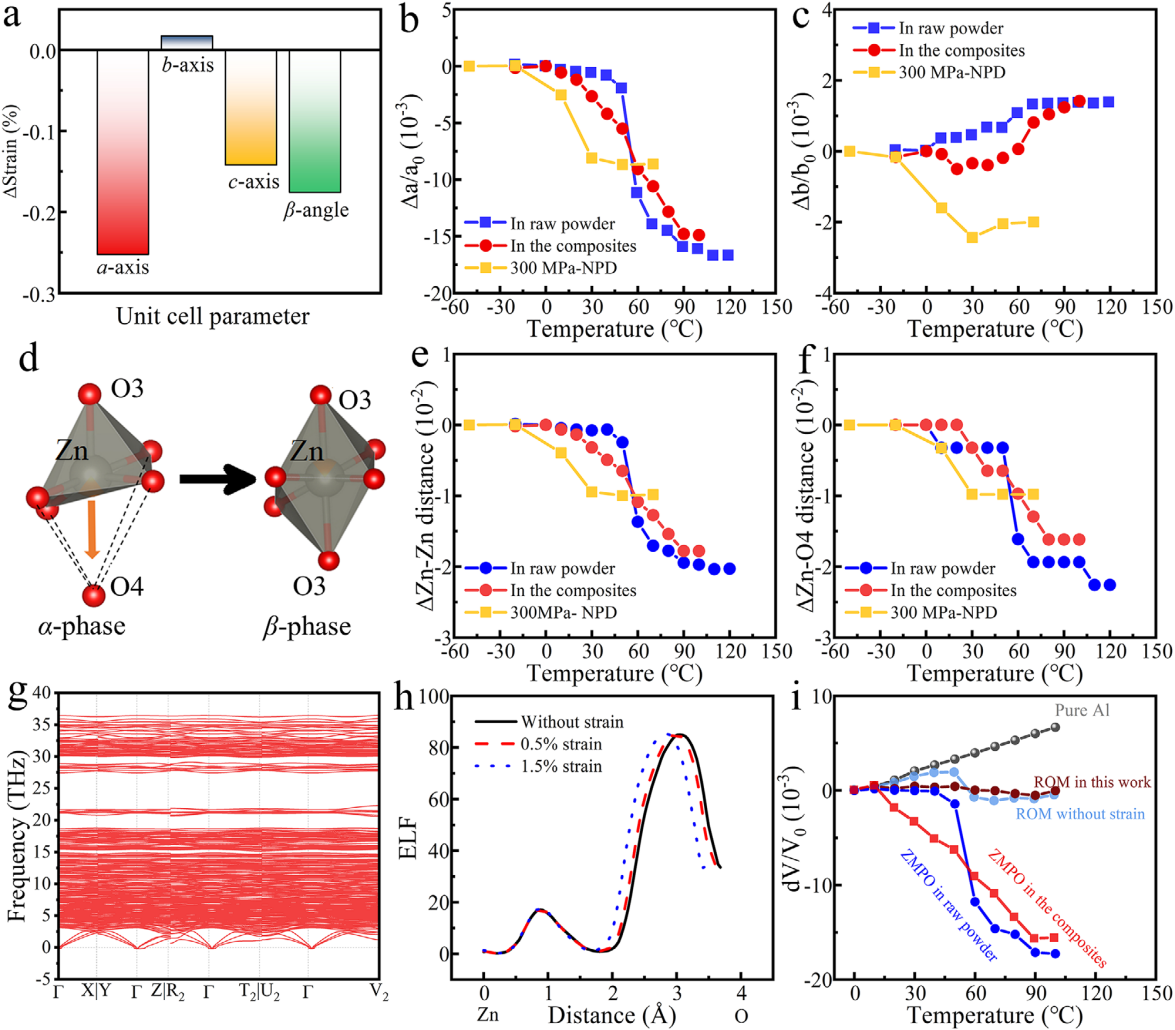
Relative evolution of crystal structure for ZMPO. (a) Lattice evolution of ZMPO in the composites. Relative evolution of the *a*‐axis (b) and *b*‐axis (c) in ZMPO at different states. (d) The ZnO5 polyhedron and ZnO6 octahedra in *α*‐phase and *β*‐phase, respectively. The variation of Zn2–Zn2 distance (e) and Zn‐O4 distance (f) with temperature. (g) The phonon spectrum of ZMPO under 0.5% compressive strain. (h) ELF one‐dimensional line chart. (i) Intrinsic unit cell CTE of ZMPO and the 35ZMPOAl composite.

Lattice strain induced in ZMPO during composite fabrication was calculated based on its room‐temperature crystal structure (Table S3), with results presented in Figure 5a. Under compressive stress from the Al matrix, ZMPO exhibits increased symmetry, manifested as reductions of 0.25%, 0.143%, and 0.177% in the *a*‐axis, *c*‐axis, and *β*‐angle, respectively. Meanwhile, the *b*‐axis expands slightly by 0.017%. To clarify the strong NTE effect of ZMPO in the composites, unit cell evolution was derived from structural refinement of NPD (Table S9) and XRD (Tables S10 and S11) data. Typically, over 0–110°C, the *a*‐axis (Figure 5b), *c*‐axis (Figure S11), and *β*‐angle of raw ZMPO decrease, while the *b*‐axis exhibits a slight expansion (Figure 5c). Intriguingly, under 300 MPa, the distinct transition temperature of the *a*‐axis shifts from 30°C to a lower value of −20°C, and the *a*‐axis decreases at a slower rate post‐pressurization. The *c*‐axis and *β*‐angle follow a similar trend (Figure S11). The *b*‐axis (Figure 5c) exhibits NTE behavior instead of PTE under 300 MPa, which may be attributed to the high pressure applied [45]. Moreover, the uniform and temperature‐advanced NTE behavior is also evident in the composites (Figure 5b). The distinct phase transition of the *a*‐axis in the composites is initiated at 0°C, a result of the lower compressive stress (160 MPa) within the composite system. Specifically, the *a*‐axis of ZMPO in the composites decreases uniformly over the broad temperature range of 0–90°C, accompanied by a substantial shrinkage magnitude. Other lattice parameters of ZMPO in the composites (Figure S11) exhibit analogous evolutionary processes. Notably, the room‐temperature unit cell volume (1448.14 Å^3^) of ZMPO (Table S3) exceeds that at 100°C (1428.414 Å^3^), retaining the basis for its strong NTE effect (Table S11). Owing to the synergistic effects of these lattice parameter variations, ZMPO in the composites undergoes a more uniform unit cell transformation (Figure 5i).

To further elucidate the ZTE mechanism of the composites, the local atomic and electronic structures of ZMPO were investigated. The variation in the interatomic distance between adjacent Zn2 atoms (Figure 1c) and the transformation of Zn‐O polyhedra (Figure 5d) that characterize the phase transition were extracted. As shown in Figure 5e, adjacent Zn2 atoms move closer more rapidly and at lower temperatures under pressure. This phenomenon arises from the reduced difference in unit cell parameters between the *α*‐phase and *β*‐phase post‐pressurization, which minimizes the required displacement of Zn atoms [44, 45]. The transformation of distorted ZnO5 polyhedra to more symmetric ZnO6 octahedra follows a similar trend (Figure 5f). In raw ZMPO powder, the Zn‐O4 interatomic distance decreases sharply by 1.62% between 50°C and 70°C, resulting in a steep NTE behavior. Intriguingly, this abrupt transition is spread evenly across 20–80°C in the composites (with the same total contraction magnitude), enabling the achievement of a broad, robust NTE effect. In terms of electronic structure, first‐principles DFT calculations (Figure S12) show that the phonon dispersion spectrum of ZMPO under 0.5% compressive strain has no imaginary frequencies (Figure 5g), confirming the stability of its monoclinic structure under pressure. Analysis of the electron localization function (ELF, Figure S13) was used to quantify the electron localization distance in Zn─O bonds under compressive strain (Figure 5h). The Zn─O interatomic distance gradually shortens with increasing compressive strain, which alleviates the distortion of Zn‐O polyhedra during the phase transition [63]. In essence, the inherent compressive strain in the composite reduces the magnitude of structural rearrangement required for the phase transition of ZMPO. This alleviates the abrupt local structural transformation of ZMPO, ultimately yielding a uniform thermal compensation effect over a broad temperature range.

Rietveld refinement (Table S10 and Table S11) of the diffraction data (Figure S10) reveals the intrinsic unit cell CTE (where dL/L_0_ = dV/3V_0_, *α*_V_ ≈ 3*α*_L_) for both raw ZMPO powder and ZMPO in 35ZMPOAl composites [3, 48]. For the NTE phase ZMPO, the raw powder exhibits a rapid volumetric contraction between 50°C and 70°C, whereas ZMPO in the composites shows a more uniform contraction over the 10–90°C range (Figure 5i). Notably, ZMPO retains a large volume shrinkage rate corresponding to an intrinsic *α*_V_ of 1.44% (corresponding to *α*_L_ = −52.5 ppm/°C) within this extended temperature window, ensuring its strong thermal compensation capability. As a result, compared to the narrow shrinkage observed in raw ZMPO powder (50–70°C), the overall *α*_V_ of the 35ZMPOAl composite with strain‐engineered ZMPO exhibits a CTE of −2.91 ppm/°C from 10°C to 90°C (corresponding to *α*_L_ = −0.97 ppm/°C). These findings confirm that strain engineering‐driven structural phase transition in ZMPO is a feasible strategy to achieve effective thermal compensation for Al matrix composites.

## 3 Conclusion

In summary, an Al matrix composite with excellent ZTE performance (25–80°C, 0.90 ppm/°C) and high thermal conductivity (80.9 W·m^−1^·K^−1^) was achieved via strain‐induced homogenization of the NTE phase transition. The NTE reinforcement exhibits a well‐coherent interface with the Al matrix. The inevitable thermal mismatch stress (160 MPa) generated during composite fabrication reaches the threshold required to trigger the phase transition of the NTE reinforcement. This pressure‐induced compressive strain modifies the phase structure of ZMPO (e.g., −0.25% along the *a*‐axis), mitigating abrupt phase transitions while maintaining efficient volumetric shrinkage of 1.44%. Specifically, under compressive strain, the interatomic distance fluctuations between neighboring Zn2 atoms and the structural evolution of Zn‐O polyhedra in ZMPO exhibit a more homogeneous progression. Leveraging this enhanced thermal compensation effect, the Al matrix composite readily achieves ZTE with a low NTE content of 35 vol.% over a broad temperature range of 55°C, in contrast to the narrow 20°C operating window of raw ZMPO. The high‐performance ZMPO/Al composites developed in this work offer an alternative perspective and feasible strategy for utilizing other abrupt yet strong NTE materials.

## 4 Experimental Methods

### 4.1 Synthesis of Samples

The synthetic Zn_1.6_Mg_0.4_P_2_O_7_ powders [51] and pure Al powders (Northeast Light Alloy Co., Ltd.) were used as raw materials. The composites were sintered by SPS (60T20KA) at 550°C under 40 MPa for 10 min. The Zn_1.6_Mg_0.4_P_2_O_7_ content in the composites varied from 20 to 40 vol.% (denoted as 20ZMPOAl‐40ZMPOAl).

### 4.2 Sample Characterization

The room‐temperature XRD patterns were tested with Co *K*α radiation (Rigaku). The temperature‐dependent XRD measurements from −20°C to 120°C were conducted with Cu *K*α radiation (PANalytical). The surface morphologies of the composites were analyzed utilizing a scanning electron microscope (GeminiSEM500), equipped with X‐ray energy dispersive spectroscopy (EDS). The atomic‐resolution images of the composites were obtained on a field‐emission TEM (FEI Tecnai G2). The vibrational modes of ZMPO in the composites were characterized using a Raman spectrometer (Senterra). Pressure‐dependent Raman spectra were recorded over a range of 0–880 MPa (Horiba) [61]. The structure refinements of XRD and NPD patterns were performed using the GSAS2 program based on the Rietveld method.

### 4.3 NPD Measurements

NPD measurements were performed on Zn_1.6_Mg_0.4_P_2_O_7_ at the High‐Pressure Neutron Diffractometer (HPND) of China Spallation Neutron Source (CSNS). During NPD measurements, samples were loaded into VNi null matrix cans with a diameter of 9.4 mm, and data were collected over a wavelength range from 0.71 to 5.8 Å. The VNi null matrix cans were selected to protect the samples against atmospheric moisture while ensuring no impact on the quality of NPD data.

### 4.4 Thermal Property Measurements

The linear thermal expansion was tested by an advanced thermal dilatometer (NETZSCH, DIL 402). The thermal conductivity was measured using a laser thermal conductivity meter (Netzsch LFA467). Additionally, samples were sprayed with a thin graphite layer on both surfaces to minimize laser reflection. All data were obtained from three independent samples, with final results presented as the average of three parallel measurements. Composite density was determined via the Archimedes water displacement method, and relative density was calculated from the theoretical and measured densities. Thermal conductivity was calculated as:

(1) $$\lambda =\alpha \;\rho \;C_{p}$$

where *α* is thermal diffusivity, *ρ* is bulk density, and *C*_p_ is specific heat capacity.

For theoretical analysis, three typical models were employed to evaluate the thermal transport behavior:

(1) Rule of Mixture (ROM) Model

(2) $$\lambda_{\mathrm{ROM}}=\left(1-V_{p}\right)\lambda_{m}+V_{p}\lambda_{p}$$

(2) Maxwell Model

(3) $$\lambda_{\mathrm{Max}}=\lambda_{m}\frac{2\left(1-V_{p}\right)\lambda_{m}+\left(1+2V_{p}\right)\lambda_{p}}{\left(2+V_{p}\right)\lambda_{m}+\left(1-V_{p}\right)\lambda_{p}}$$

(3) Hasselman–Johnson (H–J) Model

(4) $$\lambda_{H-J}=\lambda_{m}\frac{2\left(\frac{\lambda_{p}}{\lambda_{m}}--\frac{\lambda_{p}R_{k}}{r}\;-1\right)V_{p}+\frac{\lambda_{p}}{\lambda_{m}}+\frac{2\lambda_{p}R_{k}}{r}+2}{\left(1-\frac{\lambda_{p}}{\lambda_{m}}+\frac{\lambda_{p}R_{k}}{r}\right)V_{p}+\frac{\lambda_{p}}{\lambda_{m}}+\frac{2\lambda_{p}R_{k}}{r}+2}$$

where *V*_p_ is the volume fraction of ZMPO, *λ*_m_ and *λ*_p_ are the thermal conductivities of the Al matrix and ZMPO reinforcement, respectively. *r* is the average particle radius of the ZMPO, and *R*_k_ is the interfacial thermal resistance.

### 4.5 Thermal Stress Calculation

Thermal mismatch stress was measured using a micro‐area X‐ray residual stress measurement system (Rigaku AutoMate II). The residual thermal stress was calculated by the finite element method. The material parameters used in the numerical calculations were derived from the previous references [32, 34].

### 4.6 DFT Calculations

The first‐principles calculations were performed using the Vienna Ab initio Simulation Package (VASP) [64]. The projector augmented‐wave (PAW) pseudopotential was used to describe the electron‐ion interactions, and 3s^2^, 3d^10^4s^2^, 3s^2^3p^3^, and 2s^2^2p^4^ were taken as valence electrons for Mg, Zn, P, and O, respectively. The exchange‐correlation was described by the Perdew–Burke–Ernzerhof (PBE) functional within the generalized gradient approximation (GGA) [65]. The plane‐wave cutoff energy was set as 520 eV, and the k‐points were generated by the Monkhorst–Pack scheme 4 with a grid of 4×4×4. The phonon spectrum was calculated with density functional perturbation theory (DFPT). The doped structure was generated by replacing 25% Zn with Mg using the special quasi‐random structure (SQS) method. The pre‐ and post‐processes were facilitated by PHONOPY [66], VASPKIT [67], ATAT [68], and VESTA.

## Funding

This work was supported by the National Key Research and Development Program of China (No. 2025YFF0521103), the Beijing Outstanding Young Scientist Program (No. JWZQ20240101015), the National Natural Science Foundation of China (No. 52501202), and the State Key Laboratory for Advanced Metals and Materials (Grant Nos. 2025‐Z38, 2025‐Z39).

## Conflicts of Interest

The authors declare no conflicts of interest.

## Supporting information




**Supporting File**: advs75748‐sup‐0001‐SuppMat.docx.

## Data Availability

Research data are not shared.
