# Supplementary material for: Wide Temperature Zero Thermal Expansion in Al Matrix Composites with High Thermal Conductivity
Source: Adv Sci (Weinh). 2026 May 19;13(45):e75748. doi: 10.1002/advs.75748 (PMC13335785; doi:10.1002/advs.75748)
Supplement: Supplementary file 1 [file ADVS-13-e75748-s001.docx]

Supporting Information

Wide Temperature Zero Thermal Expansion in Al Matrix Composites with High Thermal Conductivity

*Jinrui Qian^†^, Feixiang Long^†^, Yiqing Liu, Longlong Fan, Mingqing Liao, Qilong Gao, Hao Lu, Dexiang Gao, Le Kang, Yuzhu Song, Xiuzhu Han, Yue Sun, Naike Shi^*^, Chang Zhou^*^, Jun Chen*

^†^Equally contributed authors

**Figure S1.** Rietveld refinement plots of XRD patterns. a) Raw ZMPO powder. b) 35ZMPOAl sample.

**Figure S2.** The crystal structure of Zn_1.6_Mg_0.4_P_2_O_7_.

**Figure S3.** SAED patterns taken from (a) the upper-right white region and (b) the lower-left gray region in Figure 2b.

**Figure S4.** EDS elemental line scan of Al, Zn, Mg, P, and O at the interface in Figure 2b.

**Figure S5.** EDS elemental mappings of the 35ZMPOAl composite.

**Figure S6.** Thermal cycling stability and reversibility of the 35ZMPOAl composite upon heating and cooling.

**Figure S7.** Thermal hysteresis behavior of the 35ZMPOAl composite during heating and cooling.

**Figure S8.** Experimental and theoretical thermal conductivities of ZMPO/Al composites.

**Figure S9.** Compressive performance of the composites. a) Compressive stress–strain curves. b) Corresponding yield strength values.

**Figure S10.** Temperature-dependent XRD patterns of ZMPO from -20 to 120 °C in a) raw powder and b) 35ZMPOAl composite.

**Figure S11.** Relative evolution in ZMPO lattice parameters with temperature for a) the *c*-axis and b) the *β*-angle.

**Figure S12.** The phonon spectrum of ZMPO under a) no pressure and b) 1.5% compressive strain.

**Figure S13.** ELF contour maps of selected planes for ZMPO under different compressive strains.

**Table S1.** Refined lattice parameters of ZMPO in the raw powder.

| Lattice parameter | *a*  (Å) | *b*  (Å*)* | *c*  (Å) | *β*  (°) | *V*  (Å^3^) | Content  (wt.%) |
| --- | --- | --- | --- | --- | --- | --- |
| *α*-ZMPO | 20.0811(6) | 8.2714(1) | 9.1110(1) | 106.262(1) | 1452.759(53) | 97.1 |
| *β*-ZMPO | 6.585(7) | 8.310(2) | 4.523(3) | 104.931(16) | 239.17(21) | 2.9 |

Note: *V_α_*_-ZMPO_=6*V_β_*_-ZMPO_.

**Table S2.** Refined lattice parameters of ZMPO in the 35ZMPOAl composite.

| Lattice parameter | *a*  (Å) | *b*  (Å*)* | *c*  (Å) | *β*  (°) | *V*  (Å^3^) | Content  (wt.%) |
| --- | --- | --- | --- | --- | --- | --- |
| *α*-ZMPO | 20.0667(18) | 8.2736(2) | 9.1068(4) | 106.214(2) | 1451.80(11) | 85.5 |
| *β*-ZMPO | 6.585(4) | 8.276(1) | 4.517(2) | 104.98(1) | 237.76(14) | 14.5 |

**Table S3.** Average lattice parameters of ZMPO in raw powder and the 35ZMPOAl composite.

| State | $\bar{a}$  (Å) | $\bar{b}$  (Å*)* | $\bar{c}$  (Å) | $\bar{\beta}$  (°) | $\bar{V}$  (Å^3^) |
| --- | --- | --- | --- | --- | --- |
| Raw powder | 20.072(1) | 8.2725(2) | 9.1091(3) | 106.223(1) | 1452.24(9) |
| Composite | 20.022(3) | 8.2739(2) | 9.0961(9) | 106.035(2) | 1448.14(2) |

**Table S4**. Thermal conductivity (*λ*)-related parameters including density *(ρ*), thermal diffusivity (*k*), and specific heat capacity (*C*p).

| Sample | *ρ*  (g/cm^3^) | *k*  (mm^2^/s) | *C*_p_ (J/(Kg·K)) | *λ*  (W·m^-1^·K^-1^) | Relative density (%) |
| --- | --- | --- | --- | --- | --- |
| 20ZMPOAl | 2.932 ± 0.028 | 62.93±0.16 | 0.764 ± 0.04 | 141.0 ± 7.5 | 97.7 |
| 25ZMPOAl | 3.015 ± 0.004 | 53.87 ± 0.39 | 0.747 ± 0.04 | 121.3 ± 6.6 | 98.0 |
| 30ZMPOAl | 3.100 ± 0.004 | 44.41 ± 0.59 | 0.722 ± 0.01 | 99.4 ± 1.9 | 98.4 |
| 35ZMPOAl | 3.193 ± 0.001 | 36.12 ± 1.20 | 0.701 ± 0.04 | 80.9 ± 5.3 | 99.0 |
| 40ZMPOAl | 3.224 ± 0.001 | 32.9 ± 0.52 | 0.681 ± 0.05 | 72.2 ± 5.4 | 97.7 |

**Table S5.** Experimental thermal conductivity of composites with different ZMPO contents and theoretical values predicted by the Rule of Mixture (ROM), Maxwell, and Hasselman-Johnson (H-J) models.

| Modeling | ROM  (W·m^-1^·K^-1^) | Maxwell  (W·m^-1^·K^-1^) | H-J  (W·m^-1^·K^-1^) | Experiment  (W·m^-1^·K^-1^) |
| --- | --- | --- | --- | --- |
| 15ZMPOAl | 189.6 | 176.4 | 176.4 |  |
| 20ZMPOAl | 178.5 | 162.3 | 162.3 | 141 |
| 25ZMPOAl | 167.3 | 148.8 | 148.8 | 121 |
| 30ZMPOAl | 156.2 | 135.9 | 135.9 | 99.4 |
| 35ZMPOAl | 145.1 | 123.6 | 123.6 | 80.9 |
| 40ZMPOAl | 134.0 | 111.7 | 111.7 | 72.2 |
| 45ZMPOAl | 122.8 | 100.4 | 100.4 |  |

**Table S6.** Parameters used for calculation of interfacial thermal resistance (*R*_k_).

| Parameters | *ρ*_Al_  (g/cm^3^) | *C*_p_  (g/cm^3^) | *ν*_Al_  (m/s) | *ρ*_ZMPO_  (g/cm^3^) | *ν*_ZMPO_  (m/s) | *R*_k_  (m^2^·K·W^-1^) |
| --- | --- | --- | --- | --- | --- | --- |
| Values | 2.7 | 0.897 | 2370 | 4 | 3866 | 0.449×10^-8^ |

**Table S7.** Comparison of thermal expansion and thermal conductivity between the ZMPO/Al composite and reported Al-matrix composites with low/near-zero/zero thermal expansion (low thermal expansion (LTE), near-zero thermal expansion (NZTE)). Listed parameters include classification category, linear thermal expansion coefficient (*α*_L_), service temperature range (Δ*T*), room-temperature thermal conductivity (*λ*), density (*ρ*), and thermal expansion test method.^[22,34,42,54-60]^

| Category | Materials | *α*_L_  (ppm/°C) | ∆*T*  (°C) | *λ*  (W·m^-1^·K^-1^) | *ρ*  (g/cm^3^) | Method (CTE) |
| --- | --- | --- | --- | --- | --- | --- |
| High PTE | Pure Al | 23.5 | 25 – 85 | 223 | 2.7 | DIL |
| LTE (3<*α*_L_≤10) | 55 wt.% Si-Al | 9.9 | 100 – 400 | 120.7 | 2.475 | DTA |
|  | 60 wt.% Si-Al | 9.92 | RT – 150 | 128 | 2.462 | DIL |
|  | 70 wt.% Si-Al | 7.5 | RT – 150 | 110.4 | 2.364 | DIL |
|  | 59.9 vol.% SiC/4032Al | 6.6 | 25 – 500 | 135 | 2.96 | DIL |
|  | 63 vol.% SiC/Pure Al | 8.21 | RT – 200 | 178 | 2.97 | DIL |
|  | 40 vol.% Cu_2_P_2_O_7_/2024Al | 8.83 | -33 – 67 | 75.9 | 3.28 | PPMS |
|  | 40 vol.% Cu_2_P_2_O_7_/AlSi | 5.56 | -150 – 75 | 65 |  | DIL |
|  | 40 vol.% ZrW_2_O_8_/AlSi | 8.81 | -98 – 52 | 61 | 3.556 | DIL |
|  | 45 vol.% ZrW_2_O_8_/AlSi | 6.94 | -98 – 52 | 47.3 | 3.668 | DIL |
|  | 50 vol.% ZrW_2_O_8_/AlSi | 5.85 | -98 – 52 | 40.7 | 3.78 | DIL |
|  | Kovar | 5.2 |  | 11-17 | 8.2 | DIL |
|  | **20 vol.%**  **ZMPO /Pure Al** | 9.88 | 25 – 85 | 141 | 2.932 | DIL |
|  | **25 vol.%**  **ZMPO/Pure Al** | 6.69 | 25 – 85 | 121 | 3.015 | DIL |
|  | 30 vol.%  Mn_3_Zn_1-x_Sn_x_N/Pure Al | 9.9 | -8 – 57 | 60 |  | DIL |
| NZTE (1≤*α*_L_≤3) | 56 vol.% Cu_2_P_2_O_7_/ZL101 | 1.73 | -33 – 32 | 31.4 |  | PPMS |
|  | 50 vol.% Cu_2_P_2_O_7_/AlSi | 2.5 | -150 – 75 | 52 |  | DIL |
|  | 64 vol.% ZrW_2_O_8_/AlSi | 1.09 | -50 – 120 |  |  | DIL |
|  | 54 vol.% ZrW_2_O_8_/AlSi | 2.05 | -50 – 120 |  |  | DIL |
|  | Invar | 1.7 | RT – 200 | 12.8 | 8.1 | DIL |
|  | **30 vol.%**  **ZMPO/Pure Al** | 2.85 | 25 – 85 | 99.4 | 3.1 | DIL |
| ZTE (*\|α_L_\|<*1) | 50 vol.%  Mn_3_Zn_1-_*_x_*Sn*_x_*N/Pure Al | 0.2 | -8 – 57 | 33 |  | DIL |
|  | 50 vol.% Cu_2_P_2_O_7_/2024Al | 0.53 | -33 – 67 | 49.7 | 3.41 | PPMS |
|  | 70 vol.%  ZrW_2_O_8_/Pure Al | 0.79 | -140 – 150 | 33 | 4.3 | DIL |
|  | **35 vol.%**  **ZMPO/Pure Al** | 0.9 | 25 – 85 | 80.9 | 3.193 | DIL |

Abbreviations: PPMS = Physical Property Measurement System, DIL = push-rod dilatometer, Differential Thermal Analysis=DTA.

**Table S8.** Phase fractions of *α*- and *β*-ZMPO in the raw powder and the 35ZMPOAl composite (XRD).

| Temperature  (°C) | *α*-ZMPO  in raw powder (wt.%) | *β*-ZMPO  in raw powder (wt.%) | *α*-ZMPO  in the composite (wt.%) | *β*-ZMPO  in the composite (wt.%) |
| --- | --- | --- | --- | --- |
| -20 | 100 | 0 | 90 | 10 |
| 0 | 100 | 0 | 90 | 10 |
| 10 | 97.1 | 2.9 | 89.3 | 10.7 |
| 20 | 96.6 | 3.4 | 87.6 | 12.4 |
| 30 | 96.1 | 3.9 | 77.8 | 22.2 |
| 40 | 93.8 | 6.2 | 68.3 | 31.7 |
| 50 | 87.6 | 12.4 | 58.4 | 41.6 |
| 60 | 31.7 | 68.3 | 35.3 | 64.7 |
| 70 | 14.5 | 85.5 | 24.2 | 75.8 |
| 80 | 11.5 | 88.5 | 10.8 | 89.2 |
| 90 | 3.6 | 96.4 | 0 | 100 |
| 100 | 2.6 | 97.4 | 0 | 100 |
| 110 | 0 | 100 |  |  |
| 120 | 0 | 100 |  |  |

**Table S9.** Average lattice parameters of ZMPO under 300 MPa at variable temperatures (NPD).

| Temperature  (°C) | $\bar{a}$  (Å) | $\bar{b}$  (Å*)* | $\bar{c}$  (Å) | $\bar{\beta}$  (°) | $\bar{\nu}$  (Å^3^) |
| --- | --- | --- | --- | --- | --- |
| -50 | 19.9109 | 8.2991 | 9.0625 | 105.48 | 1443.181 |
| -20 | 19.9115 | 8.2977 | 9.0627 | 105.484 | 1442.994 |
| 10 | 19.8605 | 8.2858 | 9.0395 | 105.155 | 1435.812 |
| 30 | 19.7498 | 8.2789 | 9.0313 | 104.998 | 1426.368 |
| 50 | 19.7379 | 8.2821 | 9.0311 | 105.004 | 1425.996 |
| 70 | 19.7390 | 8.2825 | 9.0325 | 105.013 | 1426.296 |

**Table S10.** Average lattice parameters of ZMPO in raw powder at different temperatures (XRD).

| Temperature  (°C) | $\bar{a}$  (Å) | $\bar{b}$  (Å*)* | $\bar{c}$  (Å) | $\bar{\beta}$  (°) | $\bar{\nu}$  (Å^3^) |
| --- | --- | --- | --- | --- | --- |
| -20 | 20.0820 | 8.2686 | 9.1103 | 106.283 | 1452.084 |
| 0 | 20.0793 | 8.2684 | 9.1094 | 106.278 | 1451.761 |
| 10 | 20.0728 | 8.2713 | 9.1094 | 106.2473 | 1452.00561 |
| 20 | 20.0694 | 8.2715 | 9.1091 | 106.2384 | 1451.78641 |
| 30 | 20.0673 | 8.2721 | 9.1086 | 106.2314 | 1451.72618 |
| 40 | 20.0630 | 8.2738 | 9.1064 | 106.1914 | 1451.63862 |
| 50 | 20.0401 | 8.2738 | 9.1016 | 106.1276 | 1449.69916 |
| 60 | 19.8538 | 8.2773 | 9.0599 | 105.497 | 1434.67587 |
| 70 | 19.7981 | 8.2793 | 9.0483 | 105.3025 | 1430.53558 |
| 80 | 19.7867 | 8.2795 | 9.0462 | 105.2648 | 1429.66641 |
| 90 | 19.7580 | 8.2795 | 9.0381 | 105.1838 | 1426.88653 |
| 100 | 19.7540 | 8.2797 | 9.0379 | 105.1687 | 1426.7006 |
| 110 | 19.7423 | 8.2795 | 9.0368 | 105.133 | 1425.894 |
| 120 | 19.7427 | 8.2798 | 9.0372 | 105.135 | 1426.032 |

**Table S11.** Average lattice parameters of ZMPO in the 35ZMPOAl composite at different temperatures (XRD).

| Temperature  (°C) | $\bar{a}$  (Å) | $\bar{b}$  (Å*)* | $\bar{c}$  (Å) | $\bar{\beta}$  (°) | $\bar{\nu}$  (Å^3^) |
| --- | --- | --- | --- | --- | --- |
| -20 | 20.052 | 8.2738 | 9.1050 | 106.117 | 1451.063 |
| 0 | 20.052 | 8.2751 | 9.1039 | 106.126 | 1451.186 |
| 10 | 20.041 | 8.2744 | 9.1121 | 106.061 | 1451.929 |
| 20 | 20.028 | 8.2710 | 9.0996 | 106.061 | 1448.508 |
| 30 | 19.999 | 8.2723 | 9.0937 | 105.964 | 1446.363 |
| 40 | 19.968 | 8.2719 | 9.0878 | 105.878 | 1443.729 |
| 50 | 19.942 | 8.2736 | 9.0831 | 105.787 | 1442.029 |
| 60 | 19.870 | 8.2756 | 9.0659 | 105.544 | 1437.905 |
| 70 | 19.840 | 8.2818 | 9.0619 | 105.425 | 1435.265 |
| 80 | 19.795 | 8.2837 | 9.0510 | 105.283 | 1431.62 |
| 90 | 19.756 | 8.2854 | 9.0405 | 105.154 | 1428.318 |
| 100 | 19.754 | 8.2868 | 9.0412 | 105.171 | 1428.414 |
